# Using joint models to disentangle intervention effect types and baseline confounding: an application within an intervention study in prodromal Alzheimer’s disease with Fortasyn Connect

**DOI:** 10.1186/s12874-019-0791-z

**Published:** 2019-07-25

**Authors:** Floor M. van Oudenhoven, Sophie H.N. Swinkels, Tobias Hartmann, Hilkka Soininen, Anneke M.J. van Hees, Dimitris Rizopoulos

**Affiliations:** 1 Department of Biostatistics, Erasmus Medical Center, Rotterdam, The Netherlands; 2 Danone Nutricia Research, Nutricia Advanced Medical Nutrition, Utrecht, The Netherlands; 3 German Institute for Dementia Prevention (DIDP), Saarland University, Homburg, Germany; 4 Department of Experimental Neurology, Saarland University, Homburg, Germany; 5 Department of Neurology, Institute of Clinical Medicine, University of Eastern Finland, Kuopio, Finland; 6 Neurocenter, Department of Neurology, Kuopio Hospital, Kuopio, Finland

**Keywords:** Joint model, Intervention effect, Baseline imbalance, Fortasyn, Alzheimer’s disease

## Abstract

**Background:**

Many prodromal Alzheimer’s disease trials collect two types of data: the time until clinical diagnosis of dementia and longitudinal patient information. These data are often analysed separately, although they are strongly associated. By combining the longitudinal and survival data into a single statistical model, joint models can account for the dependencies between the two types of data.

**Methods:**

We illustrate the major steps in a joint modelling approach, motivated by data from a prodromal Alzheimer’s disease study: the LipiDiDiet trial.

**Results:**

By using joint models we are able to disentangle baseline confounding from the intervention effect and moreover, to investigate the association between longitudinal patient information and the time until clinical dementia diagnosis.

**Conclusions:**

Joint models provide a valuable tool in the statistical analysis of clinical studies with longitudinal and survival data, such as in prodromal Alzheimer’s disease trials, and have several added values compared to separate analyses.

**Electronic supplementary material:**

The online version of this article (10.1186/s12874-019-0791-z) contains supplementary material, which is available to authorized users.

## Background

Alzheimer’s disease (AD) is a neurodegenerative disorder characterised by a slow progressive deterioration of cognitive capacity. The pathophysiological changes begin long before clinical manifestations of the disorder, and the disease spectrum spans from clinically asymptomatic to severely impaired [1]. The terminology of prodromal AD designates the initial mild state of cognitive impairment, whereas the dementia state represents the subsequent clinically manifest severe cognitive impairment. The specific transition between prodromal AD and the clinical diagnosis of AD dementia can be challenging [2] as AD should not be viewed with discrete and defined clinical stages, but as a multifaceted process moving along a biological and clinical continuum [1]. Given this underlying continuum, the moment of receiving the dementia diagnosis does not represent a discrete biological event. Nonetheless, having received the dementia diagnosis does indicate a certain level of disease progression. As such, the event ‘AD dementia diagnosis’ has been used in many studies that focus on risk factors, see for example: [3–5], and has obvious impact on patient care.

Prodromal AD trials frequently collect the time until clinical dementia diagnosis in combination with longitudinal patient information. These longitudinal patient information include clinical biomarkers or performance of patients in psychometric tests and can help to describe or understand disease progression. Yet, most studies dealing with longitudinal and survival (i.e., time-to-event) data analyse the data separately, mostly by relying on well-established statistical methods such as linear mixed models for longitudinal data and Cox proportional hazard models for survival data. However, a method that allows the simultaneous modelling of longitudinal measurements with a survival outcome is the joint model for longitudinal and survival data, see for example: Wulfsohn & Tsiatis (1997) [6], Henderson et al. (2000) [7], Tsiatis & Davidian (2004) [8] and Rizopoulos (2012) [9]. By combining the longitudinal and survival data into a single statistical model, joint models can account for or infer the dependencies between the two types of data. In certain situations, e.g., when it is of interest to study the association between a clinical biomarker or cognitive measure over time and the time until clinical diagnosis, a joint modelling approach is even required. More specifically, when it is of interest to study the association between a survival outcome and an endogenous time-varying covariate, such as a biomarker or another covariate measured on patients during the study, the traditional Cox model is not appropriate [10, 11]. First approaches to fit joint models have focused on the so-called two-stage methods, in which as a first step, a model is fit to the longitudinal data, and as a second step, the fitted longitudinal values are inserted in the Cox model. Many authors, such as Dafni & Tsiatis (1998) [12], Tsiatis & Davidian (2001) [13] and Sweeting & Thompson (2011) [10], have shown that the two-stage method still provides potentially biased and inefficient estimates. In comparison, the joint model simultaneously estimates the parameters in the longitudinal and survival parts of the model, for example by relying on maximum likelihood estimation.

Joint modelling is an active area in biostatistics with numerous methodological papers (within AD research, see for example: [14–17]) and has already been adopted in several clinical research fields such as cancer [18, 19] and cardiovascular disease [20, 21]. However hands-on introductions for clinicians are still limited. This paper aims to provide an introduction into the application of joint models, motivated by data from a prodromal AD trial: the LipiDiDiet trial [22].

The LipiDiDiet trial is a randomised controlled trial, with the objective of assessing the effect of medical nutrition (Souvenaid) on cognitive functioning in patients with prodromal AD. The active component of Souvenaid is Fortasyn Connect, a specific nutrient combination designed to address nutritional requirements in the presence of AD pathology [23]. In the paper on the LipiDiDiet trial’s main results, longitudinally measured variables of cognition and time to dementia diagnosis were analysed separately. In the LipiDiDiet trial the effect on the longitudinally measured primary endpoint related to cognition did not reach significance in the primary model, while in secondary models significance was reached. In addition, benefits were seen on longitudinal measures of cognition and function, and brain atrophy measures, which were secondary outcome measures in the trial [22]. A worsening of cognition is among the criteria for AD dementia diagnosis [24]. One could hypothesise that an intervention that is effective in decreasing or preventing cognitive decline would also prevent or delay the clinical diagnosis. In this paper we show how we can use joint models to optimally utilise the relationship between the longitudinal information and the event times in order to gain understanding into the process of how an intervention affects disease progression. In doing so, the application of joint models reveals relevant information about the strength and the type of the associations between the longitudinal measures of cognition and the risk of an event. Moreover, we investigate the effect of differences in baseline characteristics on study outcome. Using a joint model, we can disentangle baseline confounding from the intervention effect. Throughout the analysis of the data at hand, we aim to introduce and illustrate the major steps in a joint modelling approach for the non-statistical reader.

## Methods

### LipiDiDiet trial

The LipiDiDiet trial is a 24-month randomised, controlled, double-blind, multi-centre trial, performed at 11 study sites across different countries. The goal of the LipiDiDiet trial was to investigate the effects of Fortasyn Connect on cognition and related measures in prodromal AD patients. For this purpose, several longitudinal measures of cognitive functioning were recorded. In this paper we include two of them: the Clinical Dementia Rating sum of boxes (CDR-SB) and memory domain from a neuropsychological test battery (NTB memory domain).

The CDR-SB score reflects global clinical impression and ranges from a score of 0 to 18, with a higher score indicating a worse status. It is obtained through a semi-structured interview of patients and informants, summing scores of cognitive functioning on each of the following domain box scores: memory, orientation, judgement and problem solving, community affairs, home and hobbies, and personal care.

NTB memory domain is a composite z-score based on Consortium to Establish a Registry for AD (CERAD) 10-word list learning immediate recall, CERAD 10-word delayed recall, and CERAD 10-word recognition. A higher z-score indicates a better memory.

Individual patients’ scores were measured at baseline, where randomisation to either the test or control group took place, as well as around months 12 and 24 with an additional visit around 6 months for NTB memory domain. At each visit it was recorded whether patients had received the diagnosis of dementia. Progression to dementia was diagnosed according to criteria defined by DSM-IV, the National Institute of Neurological and Communicative Disorders and Stroke, and the AD and Related Disorders Association criteria for AD.

**Fig. 1:**
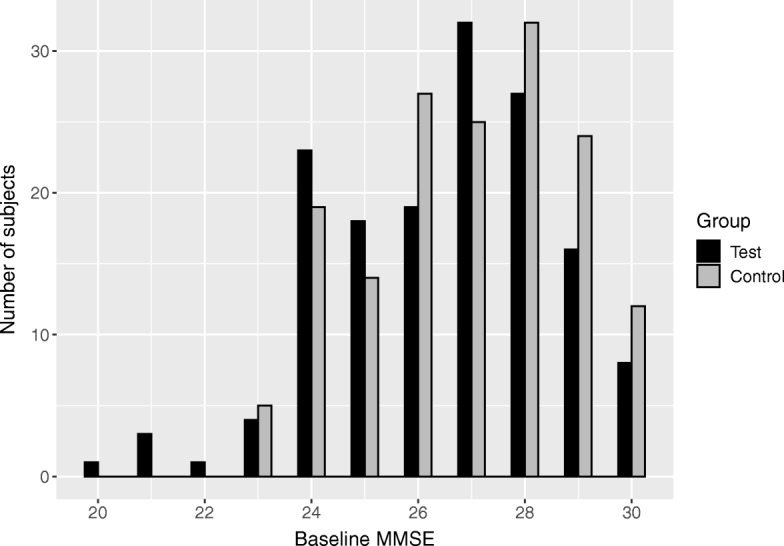
Histograms of Mini-Mental State Examination at baseline for the test and control group. The test group contains more values at the lower end of the histogram

In this article, we focus on AD dementia as a specific form of dementia. The study sample consisted of 311 patients (modified intention-to-treat population in the LipiDiDiet main paper [22]), of whom 57 (36%) patients in the control group and 62 (41%) in the test group had received the AD dementia diagnosis. The median follow-up times were respectively 1.96 years in the control, and 1.94 years in the test group. Despite the randomisation procedure, a statistically significant difference between the intervention groups was found in baseline Mini–Mental State Examination (baseline MMSE, p=0.039, two-sided t-test), reflecting baseline cognitive performance. The higher baseline MMSE score in the control group denotes better performance and suggests a lower risk of receiving the dementia diagnosis in this group at baseline. Figure 1 displays the histograms of baseline MMSE scores in the test and control group. For further information regarding the LipiDidiet trial, including information on the randomisation procedure, we refer to the LipiDiDiet main paper [22].

### Methodology for the standard joint model

As the name suggests, a joint model for longitudinal and survival data consists of a longitudinal sub-model and a survival sub-model. The longitudinal sub-model is typically a mixed effects model aiming to describe the shapes of the patient-specific longitudinal profiles. For continuous longitudinal data, linear mixed models can take into account that repeated measurements from the same patient may be more correlated than measurements from other patients, by including not only fixed effects but also patient-specific random effects. For background information on mixed models, we refer to Verbeke & Molenberghs (1997) [25] and Fitzmaurice et al. (2008) [26].

**Fig. 2:**
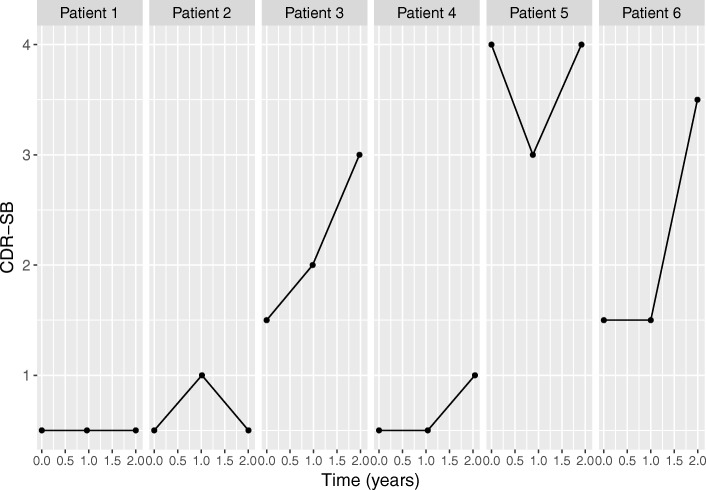
Observed longitudinal profiles for CDR-SB for six randomly selected patients. A higher CDR-SB score indicates a worsening of a patient’s status

**Fig. 3:**
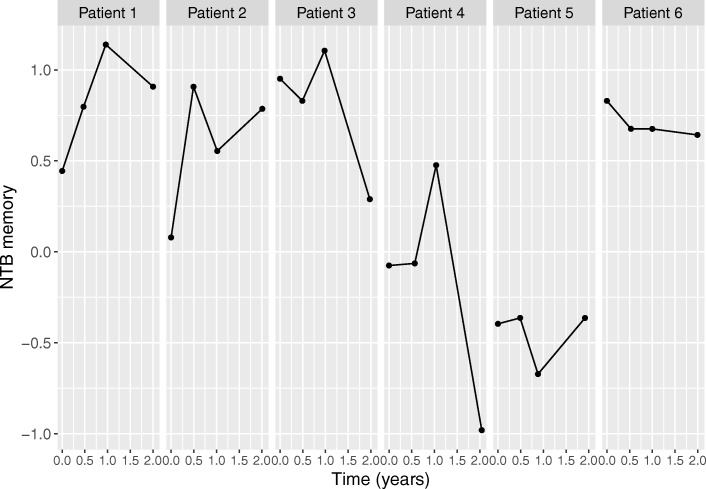
Observed longitudinal profiles for NTB memory domain for six randomly selected patients. A lower NTB memory domain score indicates a worsening of a patient’s status

In order to formulate our longitudinal sub-model for the longitudinal trajectories, as a first step we investigated the observed longitudinal profiles for six randomly selected patients. Figures 2 and 3 show the longitudinal profiles for respectively their CDR-SB and NTB memory domain observations; these figures show that there is a lot of variation between patients. Therefore, we allowed each patient to have its own trajectory, by incorporating patient-specific intercepts and slopes. For the average CDR-SB and NTB memory domain trajectories we used linear effects of time (*β*_1_) but more complicated functions of time such as quadratic or higher order polynomials, e.g., using splines, are also possible [27]. We also tried trajectory functions for CDR-SB and NTB memory domain using quadratic time effects, but these were found to give similar results (results not shown). To model the effect of Fortasyn Connect, we included both a main effect of the intervention (*β*_2_) and an interaction of intervention by time (*β*_3_) in order to allow the trajectories of the intervention groups to be different over time. This is necessary, because the intervention is expected to have a gradual effect, possibly resulting in CDR-SB and NTB-memory domain levels for the test group that are worsening more slowly. Further, we included and intercept (*β*_0_) and main effects for baseline MMSE (*β*_4_) and site (*β*_5_). This resulted in the following longitudinal sub-model for the CDR-SB observations, and similarly defined for NTB memory domain

$${}\begin{aligned} \text{CDR}_{i}(t) &= \tilde{\text{CDR}}_{i}(t) + \varepsilon_{i}(t), \\ \tilde{\text{CDR}}_{i}(t) &\,=\, \beta_{0} + \beta_{1} t + \beta_{2}\text{\texttt{fortasyn}}_{i} \!+ \beta_{3}\text{\texttt{fortasyn}}_{i} \times t\\ &~~~+ \beta_{4} \text{\texttt{bmmse}}_{i} + \beta_{5} \text{\texttt{site}}_{i} + b_{i0} + b_{i1} t, \\ \end{aligned} $$

where CDR_*i*_(*t*) are the observed values of CDR-SB for patient *i* at actual time points *t*, and the time points at which measurements take place may vary between patients. Further *b*_*i*0_ and *b*_*i*1_ denote respectively the patient-specific intercept and slope. The longitudinal profile of observed values CDR_*i*_(*t*) is broken down in a trajectory function $\tilde {\text {CDR}}_{i}(t)$ and a random error term *ε*_*i*_(*t*), which is assumed to be normally distributed. The trajectory function is assumed to describe the true but unobserved trajectory of the longitudinal marker, and as will be seen later, is used in the survival sub-model ‘joining’ the two sub-models. The main effect of the intervention, *β*_2_, denotes the difference between the intervention groups at baseline, while the interaction effect *β*_3_, describes the intervention effect over time.

**Fig. 4:**
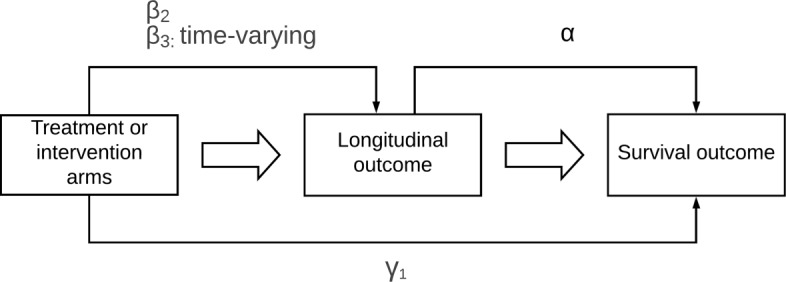
Schematic representation of a joint model. *β*_2_ and *β*_3_ denote the constant respectively time-varying *indirect* intervention effect on the longitudinal marker, *α* is the effect of the longitudinal marker on the survival outcome and *γ*_1_ is the *direct* effect on the survival outcome

A common choice for the survival sub-model is a Cox model, which is used to model the hazard of experiencing the event, i.e., in this case receiving the dementia diagnosis. For background information on Cox models, see Cox (1972) [28], Klein & Moeschberger (1997) [29] and Therneau & Grambsch (2013) [30]. In case the proportional hazard assumption of the Cox model is violated, alternative modelling frameworks for the survival sub-model exist, such as the accelerated failure time model [31]. In this paper we formulated our joint model using a Cox model. Additionally, we fitted a joint model using an accelerated failure time model which gave similar findings (results not shown). In the Cox model we included the intervention as a time-independent effect, and the estimated true trajectory of the longitudinal marker as a time-varying effect. Since there can be variation across sites in how early a patient is diagnosed, we also corrected for site in the survival sub-model. The hazard *λ*_*i*_(*t*) of dementia diagnosis at time *t* for patient *i* is therefore modeled using the following survival sub-model,

$${}\lambda_{i}(t) = \lambda_{0}(t) \exp \{ \gamma_{1}\text{\texttt{fortasyn}}_{i} + \gamma_{2} \text{\texttt{site}}_{i} + \alpha \tilde{\text{CDR}}_{i}(t) \}, $$

where the parameter *α* links the longitudinal process, i.e., the trajectory function $\tilde {\text {CDR}}_{i}(t)$, or similarly $\tilde {\text {NTB}}_{i}(t)$, to the survival process. More specifically, the quantity exp(*α*) denotes the hazard ratio at time *t* for a one-unit increase in the trajectory of the longitudinal marker at the same time point. Further, *λ*_0_(*t*) is the baseline hazard and *γ*_1_ denotes a direct effect on the survival outcome. To gain a better understanding of how the intervention affects the risk of receiving the dementia diagnosis, and to explain what we mean by a ‘direct effect’, we distinguish three types of coefficients. These are schematically illustrated in Fig. 4. *β* describes the intervention effect on the longitudinal marker. As indicated before, there are two types of *β’s* here; *β*_2_ denoting the difference in the longitudinal outcome between the intervention groups at baseline, and *β*_3_ describing the intervention effect on the longitudinal outcome over time. Secondly, since the parameter *α* measures the effect of the longitudinal process on the survival outcome, together, *β*_3_ and *α*, quantify the time-varying intervention effect on the risk of receiving the dementia diagnosis manifesting through the longitudinal marker. The third type of parameter involving the intervention is *γ*_1_ and is directly related to the risk of receiving the dementia diagnosis. Within the joint model we can therefore distinguish the direct process (Fig. 4, bottom arm), capturing the direct effect on the survival outcome, and the indirect process (Fig. 4, upper arm), in which the coefficients quantify the indirect intervention effect on the survival outcome through the longitudinal marker.

As is the case for the Cox model, the intervention effect in the joint model is the hazard ratio of the test versus the control group. In particular, the total intervention effect is the hazard ratio between two generic patients, *i* in the test group (fortasyn_*i*_=1) and *i*^′^ in the control group ($\text {\texttt {fortasyn}}_{i'} = 0\phantom {\dot {i}\!}$) who do not further differ concerning other covariates. In the joint model, this hazard ratio is a combination of the indirect and direct process. For our formulated joint model the hazard ratio for the total intervention effect denotes exp{*γ*_1_ +*α*(*β*_2_+*β*_3_×*t*)}, with the first part (i.e., *γ*_1_), for the direct process and the latter (time-varying) for the indirect process.

### Methodology for investigating the baseline confounding

The two processes of the joint model differ in how they handle the aspect of time. The indirect process can model how an intervention effect varies over time by modelling the intervention effect in the mixed model as a divergence of trajectories. In the direct process however, we are dealing with the proportional hazard assumption of the Cox regression model meaning that the direct effect on the survival outcome is assumed to be constant over the whole period. This arm is therefore likely to capture the effects already present right at the start of the intervention period. In a situation such as this one, where the effect of the intervention on the survival outcome - manifesting through the longitudinal marker - is expected to increase gradually over time, but an effect of any possible baseline confounding on the survival outcome is expected to be immediate, the baseline confounding will for a large extent end up in the direct arm of the model. This is a very appealing property of the joint model that makes it a very effective tool to investigate and control for the effect of potential baseline confounding.

MMSE, found to be significantly different at baseline, is noted to be an important predictor for outcome parameters [22]. This suggests that, before the start of the intervention, the test group might on average have been more likely to receive the dementia diagnosis than the control group due to an imbalance of baseline characteristics. This hampers the interpretability of the results as post baseline outcomes are a combination of the intervention effect and the effect of differences already present at baseline. To investigate this, we examined whether the lower baseline MMSE scores in the test group were related to higher risks of receiving the dementia diagnosis at the start of the trial, therefore possibly counteracting the intervention effect. This required fitting an additional joint model in which we corrected for the effect of the baseline MMSE score on dementia diagnosis by including its value in the survival sub-model, given by

$$\begin{array}{*{20}l} {}\lambda_{i}(t) &= \lambda_{0}(t) \exp \{ \gamma_{1}\text{\texttt{fortasyn}}_{i} + \gamma_{2}\text{\texttt{bmmse}}_{i} + \gamma_{3} \text{\texttt{site}}_{i}\\ &\quad+ \alpha \tilde{\text{CDR}}_{i}(t) \}. \end{array} $$

We will illustrate how the coefficients of this extended joint model can be an effective tool to investigate and control for the effect of potential baseline confounding using the CDR-SB data from the LipiDiDiet trial.

Naturally, apart from being a useful property in investigating possible baseline confounding, the combination of an immediate (direct) and a progressive (indirect) effect helps us to understand the process by which the intervention affects the risk of dementia diagnosis.

### Methodology for investigating the association between the longitudinal and survival process

Another aspect of the process by which the intervention affects the timing of dementia diagnosis is determined by the type of the association between the longitudinal and the survival process. The joint model defined in the previous section is the standard joint model and assumes that the value of the longitudinal outcome at any time *t* is related to the risk of an event at the same time point. However, the underlying relationship between the two processes could have a more complex nature. Examples of longitudinal characteristics possibly related to dementia diagnosis, are the current value, the stability at the current moment, the history of the longitudinal profile up to now or combinations of these characteristics [9]. For demonstration purposes we compared joint models that vary with respect to the type of association that is assumed between the longitudinal data i.e, NTB memory domain, and the survival process i.e., timing of dementia diagnosis. We investigated whether, given the current value of NTB memory domain, the rate of change (i.e., the slope) contains any additional information on the risk of receiving a dementia diagnosis. More specifically, the slope indicates by how much the NTB memory domain for a particular patient is increasing or decreasing at a specific time point. This required fitting a joint model in which we included the slope of NTB memory domain as an additional term in the survival sub-model, given by

$$\begin{array}{*{20}l} {}\lambda_{i}(t) &= \lambda_{0}(t) \exp \{ \gamma_{1}\text{\texttt{fortasyn}}_{i} + \gamma_{2}\text{\texttt{bmmse}}_{i} + \gamma_{3} \text{\texttt{site}}_{i} \\& \quad+ \alpha_{1} \tilde{\text{NTB}}_{i}(t) + \alpha_{2} \text{\texttt{slope}}_{i}(t) \}, \end{array} $$

where the slope of NTB memory domain is obtained by taking the derivative of the trajectory function, consisting of the fixed and random effects, that is,

$${}\text{\texttt{slope}}_{i}(t) = \frac{d}{dt}{\tilde{\text{NTB}}}_{i}(t) = \beta_{1} + \beta_{3} \text{\texttt{fortasyn}}_{i} + b_{i1}. $$

The parameter *α*_1_ has the same interpretation as the parameter *α* before, and the parameter *α*_2_ measures the association between the slope of the NTB memory domain trajectory and the risk of an event at the same time point, holding $\tilde {\text {NTB}}_{i}(t)$ constant. Using this joint model, two patients with the same level of NTB memory domain at the current moment do not necessarily have to be at equal risk of receiving the dementia diagnosis. For example, if one patient’s NTB memory domain level is decreasing very rapidly while another patient’s NTB memory domain level is remaining constant, it might be more realistic to assume that the first patient has a higher risk of receiving the dementia diagnosis than the latter - although they have the same value at the current moment.

A similarity between this joint model and the standard joint model, is that the risk of an event at the current moment is related to characteristics of the trajectory at that same time point only. However, the risk of receiving the dementia diagnosis may not depend solely on the level of NTB memory domain or its rate of change at the current moment, but it might also be related to the history of the NTB memory domain levels. That is, two patients with the same characteristics at the current moment are not necessarily at the same risk of receiving a dementia diagnosis if their history of NTB memory domain levels were very different. One approach to take the history of NTB memory domain levels into account is by summarising its cumulative effect i.e., the area under the curve (AUC). The area under the curve indicates the cumulative effect of NTB memory domain values for a particular patient up to the current time point. We also investigated this type of association by fitting a joint model with the following survival sub-model

$$\begin{array}{*{20}l} {}\lambda_{i}(t) &= \lambda_{0}(t) \exp \{ \gamma_{1}\text{\texttt{fortasyn}}_{i} + \gamma_{2}\text{\texttt{bmmse}}_{i} + \gamma_{3} \text{\texttt{site}}_{i}\\ &\quad+ \alpha_{3}\text{\texttt{AUC}}_{i}(t) \}, \end{array} $$

where *α*_3_ measures how strongly the risk of an event at time *t* is related to the cumulative effect of NTB memory domain for patient *i* by time point *t*. A possible limitation of this joint model is that it gives all past values of NTB memory domain the same weight in terms of their impact on the risk of receiving the dementia diagnosis at the current time point. This may not always be a reasonable assumption. As an alternative, a weight function can be used that places different weights at different time points, for example to give more weight to more recent values of the longitudinal marker. For information on how to use this weight function we refer to [9].

**Fig. 5:**
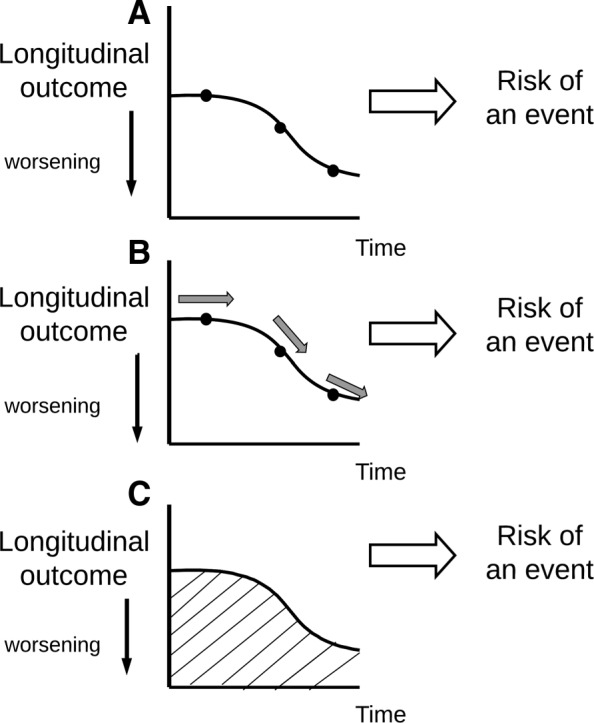
Graphical representation of different ways of modelling the association between the longitudinal and survival process. The different graphs respectively denote the current value (**a**), the current value plus the rate of change (**b**) and the cumulative effect (i.e., the AUC) of the longitudinal trajectory (**c**)

Figure 5 gives a graphical representation of different ways of modelling the association, respectively using the current value, the current value plus the rate of change and the cumulative effect of the longitudinal trajectory.

All the statistical analyses in this paper were performed with statistical software package R, using R-package **JM** [32]. The package uses maximum likelihood for the parameter estimation and assumes right-censoring. The R code to fit the joint models can be found in the web appendix (Additional file 1).

## Results

Note that results in this paper can to some extent differ from results in the LipiDiDiet main paper [22], since different types of models are used. In the main paper, mixed models were used that included the outcome baseline value as a covariate, according to a prespecified statistical analysis plan. In the results presented below, the mixed model approach is part of the joint models and in this mixed model approach, the outcome baseline values are included in the longitudinal trajectory. Modelling outcome baseline values as part of the trajectory is preferred in the joint model context as it maximises the amount of information that is used to estimate the association between the longitudinal data and the survival data.

### Results of the standard joint model

Parameter estimates, standard errors, and associated *p*-values for the standard joint model are presented in Tables 1 and 2a, respectively for CDR-SB and NTB memory domain. Not surprisingly, from the longitudinal sub-models we observe that the CDR-SB and NTB memory domain scores significantly worsen over time, reflected by an increase of on average 0.61 (95% CI: 0.52-0.70) per year for CDR-SB and a decrease of on average 0.10 (95% CI: 0.04-0.16) per year for NTB memory domain. For the CDR-SB score, however, we see that there is significantly less worsening over time in the test group than in the control group, with the average increase being 0.23 (95% CI: 0.10-0.37) per year less in the test group compared to the control group.

**Table 1:** Results for the standard joint model for CDR-SB

	a) Without baseline MMSE		b) With baseline MMSE	
	Coefficient (SE)	*p* Value	Coefficient (SE)	*p* Value
Longitudinal sub-model:				
*Time* *β*_1_	0.609 (0.047)	0.000	0.605 (0.047)	0.000
*Fortasyn* *β*_2_	0.071 (0.077)	0.361	0.077 (0.077)	0.321
*Time × Fortasyn* *β*_3_	-0.233 (0.069)	0.001	-0.234 (0.069)	0.001
*Bmmse* *β*_4_	-0.122 (0.015)	0.000	-0.110 (0.016)	0.000
	Log Hazard (SE)	*p* Value	Log Hazard (SE)	*p* Value
Survival sub-model:				
*Fortasyn* *γ*_1_	0.394 (0.204)	0.053	0.125 (0.210)	0.553
*Ass.* *α*	0.701 (0.077)	0.000	0.664 (0.083)	0.000
*Bmmse* *γ*_2_	-	-	-0.228 (0.050)	0.000

**Table 2:** Results for the different types of joint models for NTB memory domain

	a) Current value		b) Current value plus slope		c) Cumulative effect	
	Coefficient (SE)	*p* Value	Coefficient (SE)	*p* Value	Coefficient (SE)	*p* Value
Longitudinal sub-model:						
*Time* *β*_1_	-0.101 (0.030)	0.001	-0.128 (0.030)	0.000	-0.092 (0.029)	0.002
*Fortasyn* *β*_2_	0.042 (0.082)	0.617	0.039 (0.083)	0.640	0.042 (0.083)	0.610
*Time × Fortasyn* *β*_3_	0.052 (0.043)	0.219	0.049 (0.043)	0.254	0.054 (0.042)	0.192
*Bmmse* *β*_4_	0.160 (0.021)	0.000	0.159 (0.021)	0.000	0.158 (0.021)	0.000
	Log Hazard (SE)	*p* Value	Log Hazard (SE)	*p* Value	Log Hazard (SE)	*p* Value
Survival sub-model:						
*Fortasyn* *γ*_1_	0.154 (0.203)	0.449	0.513 (0.387)	0.185	0.112 (0.198)	0.573
*Ass.* *α*_1_	-1.214 (0.174)	0.000	-1.162 (0.341)	0.001	-	-
*Ass. slope* *α*_2_	-	-	-6.792 (1.854)	0.000	-	-
*Ass. AUC* *α*_3_	-	-	-	-	-0.671 (0.118)	0.000
*Bmmse* *γ*_2_	-0.098 (0.057)	0.085	-0.156 (0.088)	0.078	-0.163 (0.055)	0.003

Further, we observe that both scores have strong associations with the risk of receiving the dementia diagnosis. In particular, a unit increase in CDR-SB corresponds to a exp(*α*) = 2.0-fold increase (95% CI: 1.7-2.3), and a 0.2 unit decrease in NTB memory domain corresponds to a exp(−*α*×0.2) = 1.3-fold increase (95% CI: 1.2-1.4) in the risk of receiving the dementia diagnosis. Thus, as expected, high values for CDR-SB and low values for NTB memory domain are associated with higher risks of receiving the dementia diagnosis. Note that the association for NTB memory domain (z-score) is reported per 0.2-unit increase, instead of per 1 unit, since the former denotes a more realistic increase.

### Results investigating the baseline confounding

We notice from the results in Table 1a that the coefficients *β*_3_ and *α*, which together quantify the indirect intervention effect, are both significant. These results suggest that the intervention decreases the risk of receiving the dementia diagnosis through its effect on CDR-SB. Simultaneously, not surprisingly given the baseline imbalance, we observe a nearly significant direct effect with the test group being exp(*γ*_1_)=1.5-fold more likely to receive the dementia diagnosis than the control group. As explained above, the direct effect measures a constant effect over time, due to the proportional hazard assumption of the survival sub-model, and is therefore likely to capture possible effects of the baseline confounding. The significant direct effect in favour of the control group is therefore an indication of baseline confounding, also supported by the baseline difference in MMSE.

**Fig. 6:**
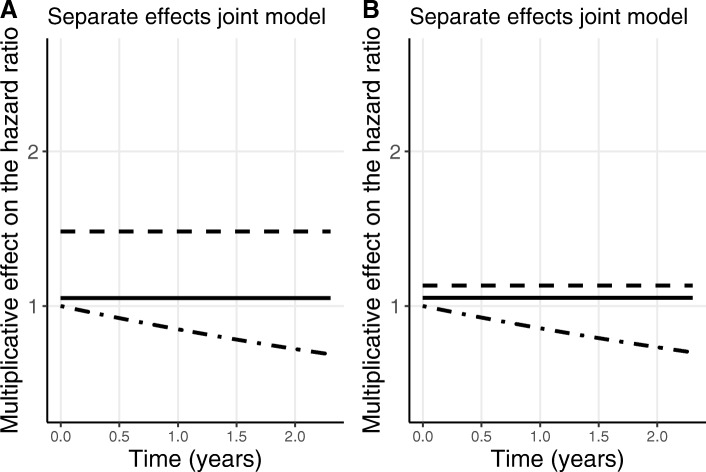
Separate effects as estimated by the joint model for CDR-SB. The separate components exp(*γ*_1_) (direct effect; dashed line), exp(*α*×*β*_2_) (indirect constant effect; solid line) and exp(*α*×*β*_3_×*t*) (indirect time-varying effect; dot dashed line), that together form the hazard ratio for the total intervention effect as estimated from the joint model for CDR-SB, plotted as separate effects in **a** without and in **b** with correction for baseline MMSE in the survival sub-model

**Fig. 7:**
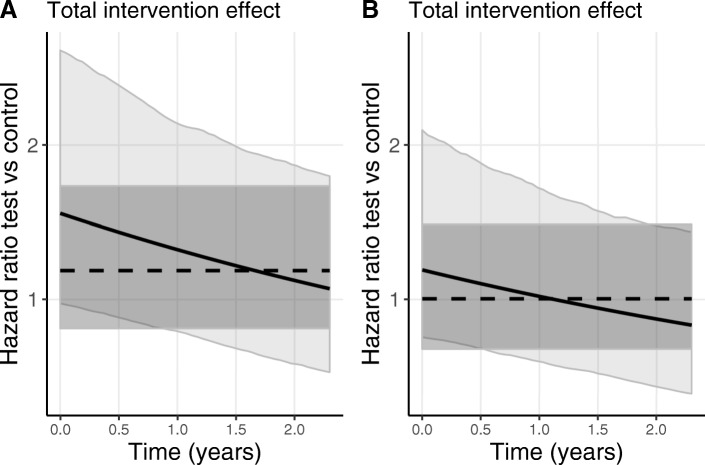
Total intervention effect as estimated by the joint model for CDR-SB. The total intervention effect on the hazard of dementia diagnosis as estimated from the joint model (solid line) and Cox model (dashed line) for CDR-SB, in **a** without and in **b** with correction for baseline MMSE in the survival sub-model. Corresponding 95% percentile confidence bands (light grey corresponding to the joint model and dark grey corresponding to the Cox model) were based on 2500 bootstrap samples

Comparing the results for *γ*_1_ of the model with (Table 1b) versus the model without baseline MMSE correction (Table 1a), we observe that by correcting for baseline MMSE in the survival sub-model, the direct effect shrinks. This is also illustrated in Fig. 6 where the effects of the coefficients for the separate components of the joint models are displayed over time. Comparing the effects of exp(*γ*_1_) (the dashed lines) for 6a versus 6b shows that including the baseline MMSE correction, made the estimate for the direct effect shift towards a hazard ratio of 1, meaning no difference. Table 1 and Fig. 6 also show that the estimates for the indirect effect components (*β*_2_, *β*_3_ and *α*) are hardly affected by the in - or exclusion - of baseline MMSE in the survival sub-model. Based on these results, we hypothesise that the baseline confounding in MMSE is indeed directly related to dementia diagnosis and that it masks the total intervention effect, being a combination of the indirect and direct processes. The latter is graphically illustrated in Fig. 7, in which the total intervention effect from the joint model - that is, the combination of the separate components of Fig. 6 - is displayed as a solid line.

Figure 7 also shows the hazard ratios for the intervention effect on dementia diagnosis as estimated from a separately run Cox model (dashed lines). We observe that by using a joint model, and more specifically by incorporating the increasing intervention effect on the longitudinal marker, we can model an increasing intervention effect over time on the risk of dementia diagnosis. While by using the (standard) Cox model, with the underlying proportional hazard assumption, the intervention effect is assumed to be constant over time from baseline onward.

### Results for the association between the longitudinal and survival process

Table 2 presents the results of joint models using the current value plus slope (b), and the cumulative effect (c) of the NTB memory domain trajectory for the link between the two processes. From the two types of joint models we observe similar results on the longitudinal process. From the association parameters, we observe that, as expected, decreasing trajectories and small cumulative values for NTB memory domain are associated with higher risks of receiving the dementia diagnosis. Both the rate of increase and the cumulative effect are strongly associated with the risk for dementia diagnosis. For example, if a patient’s NTB memory domain score decreases by 0.2 units faster per year, or 1/60 units faster per month, then the risk of dementia diagnosis is associated with a exp(−*α*_2_×0.2) = 3.9-fold (95% 1.9-8.0) increase in the hazard. In the same way, if the cumulative effect of the history of the NTB memory domain levels (i.e., AUC) decreases with one unit, then this corresponds to a exp(−*α*) = 2.0-fold increase (95% CI: 1.6-2.5) in the risk of dementia diagnosis.

We compared the two alternative types of joint models with the standard joint model based on measures for the model fit (information criteria AIC and BIC). Both measures indicated that the joint model using the current value plus slope is the best fitting joint model, suggesting that inclusion of the slope of the NTB memory domain trajectory improves the fit of the model compared to the standard joint model. The model using a cumulative effect was not found to have a better fit to the data than the standard joint model.

## Discussion

Scientists within the (prodromal) AD research field have much to gain from joint models for longitudinal and survival data. When estimating the time until clinical dementia diagnosis, while accounting for the effect of a longitudinal biomarker or cognitive measure, joint models can not only provide estimates for their association, but they can also further investigate the type of association.

This paper aimed to provide an introduction into the application of joint models with special interest in the relationship between the longitudinal information and the event times, using data from a prodromal AD trial. First of all, we reanalysed the data, combining the longitudinal data on cognitive functioning with the survival data on dementia diagnosis in order to account for their dependencies. Both longitudinal outcomes, CDR-SB and NTB memory domain, were strongly associated with the risk of dementia diagnosis. For CDR-SB we observed a statistically significant intervention effect on the longitudinal trajectory. Secondly, for NTB memory domain we investigated the type of association between the longitudinal profiles and the risk of dementia diagnosis. Specifically, we investigated three association types: the current value, the current value in combination with the rate of increase and the cumulative effect. We concluded that it was the current value in combination with the rate of increase of the longitudinal trajectory, that best captures the association with dementia diagnosis.

Additionally, this paper demonstrated the added value of a typical characteristic of joint models, namely the combination of the direct and indirect processes, both with different possibilities in modelling the effect of time. The joint model suggested an increased hazard ratio for the test versus the control group at the beginning of the trial. Given that there was no intervention before or at baseline, this increased hazard ratio was hypothesised to be caused by an imbalance between the intervention groups in characteristics at baseline. The groups were found to have a statistically significant imbalance at baseline in MMSE. MMSE is known to reflect cognitive performance and having an imbalance in MMSE between the intervention groups at baseline suggested that the groups - despite the randomisation process - might have on average differed in where they were in the disease continuum at baseline. Including baseline MMSE in the joint model markedly decreased the hazard ratio at the beginning of the trial, which fits into the hypothesis that the increased hazard ratio at the beginning of the trial was caused by baseline imbalance.

The imbalance between the intervention groups in characteristics at baseline might have been composed of several factors for which baseline MMSE was only a proxy. However, including the baseline MMSE in the joint models provided a tool to disentangle baseline confounding from the intervention effect.

Further, this paper illustrated another positive feature of joint models which is to model intervention effects on the hazard ratio that are changing over time. In the standard Cox models, the intervention effect is assumed to be constant during the entire follow-up, an assumption that is often not biologically meaningful. Using a joint model, it is possible to model a time-varying intervention effect on the survival outcome by incorporating a time-varying intervention effect on the longitudinal marker. In this prodromal AD trial, the joint model revealed an indication of an increasing intervention effect over time, suggesting a decreased hazard ratio for the test group at the end of the 24-month trial.

Using time to dementia diagnosis as an outcome measure within the limited time-frame of a clinical trial has practical issues which complicate its use. First, a large part of the diagnoses cluster around the study visits when cognitive testing is performed and progression to dementia is thus detected. As a consequence, a part of the observed event times is interval-censored, although the statistical software used for the analyses, did not cover this type of censoring. Another aspect is that, the diagnosis represents a single time point when the disease is thought of as a process moving along a continuum. Time to dementia diagnosis provided therefore only a rough measure of disease progression. However, using the information on time to dementia diagnosis was found to have an added value, by applying a statistical approach that combines every patient’s moment of diagnosis with his or her longitudinal trajectory.

## Conclusion

Joint models provide a valuable tool in the statistical analysis of clinical studies with longitudinal and survival data, such as in prodromal Alzheimer’s disease trials, and have several added values compared to separate analyses.

## Additional file

Additional file 1 R code to fit joint models. (PDF 35 kb)

## Data Availability

The data are proprietary information of the LipiDiDiet clinical study group.

## Abbreviations

AD: Alzheimer’s disease
AUC: Area under the curve
CDR-SB: Clinical dementia rating sum of boxes
CERAD: Consortium to establish a registry for AD
MMSE: Mini–mental state examination
NTB: Neuropsychological test battery
