# Supplementary material for: Using joint models to disentangle intervention effect types and baseline confounding: an application within an intervention study in prodromal Alzheimer’s disease with Fortasyn Connect
Source: BMC Med Res Methodol. 2019 Jul 25;19:163. doi: 10.1186/s12874-019-0791-z (PMC6659198; doi:10.1186/s12874-019-0791-z)
Supplement: Supplementary file 1 — R code to fit joint models. (PDF 35 kb) [file 12874_2019_791_MOESM1_ESM.pdf]

Additional file 1.

```
# As an example we will show how to fit the joint models for NTB memory domain
# that are presented in this paper

# R code for longitudinal sub-model
# We load the library needed to fit a mixed effects model
library(nlme)
lmmoFit <- lme(ntb ~  site + blmmse + timevisit*trt, data = longdata,
               random = ~ timevisit|subjid, control = lmeControl(opt = "optim"))

# R code for survival sub-models
# For the Cox model we need a dataset that only contains the survival data
# i.e., a single row per subject with the time of AD diagnosis
# and a status indicator (0 if censored, 1 if diagnosed with AD)
dat.id <- longdata[!duplicated(longdata$subjid), ]

# We load the library needed to fit a Cox model
library(survival)
coxFit <- coxph(Surv(timeAD, statusAD) ~ trt + site + blmmse, x = TRUE,
          data = dat.id)

# R code for joint models
# We load the library needed to fit a joint model
library(JM)

# We fit the standard joint model
jointFit   <- jointModel(lmmoFit, coxFit,  timeVar = "timevisit", iter.EM = 250,
                         method = "piecewise-PH-aGH")
# We use summary to obtain an output of the fitted joint model including
# measures for the model fit (AIC and BIC)
summary(jointFit)

# R code for joint models using different association structures
# To fit a joint model using the current value plus the rate of change (slope)
# we first need to specify the slope
dForm <- list(fixed = ~ 1 + trt, indFixed = c(8, 10), random = ~ 1, indRandom = 2)
jointFit.slope  <- jointModel(lmmoFit, coxFit,  timeVar = "timevisit",
                              method = "piecewise-PH-aGH", parameterization = "both",
                              derivForm = dForm, iter.EM = 250)
summary(jointFit.slope)

# To fit a joint model using the cumulative effect we first need to specify the AUC
iform <- list(fixed = ~ -1 + timevisit
              + I(timevisit * (site == "02"))
              + I(timevisit * (site == "03"))
              + I(timevisit * (site == "04"))
              + I(timevisit * (site == "08"))
              + I(timevisit * (site == "12"))
```

```
                + I(timevisit * blmmse)
                + I(timevisit^2/2)
                + I(timevisit * (trt == "2"))
                + I(timevisit^2/2 * (trt == "2")),
                indFixed = 1:10,
                random = ~ -1 + timevisit + I(timevisit^2/2), indRandom = 1:2)

jointFit.cum <- jointModel(lmmoFit, coxFit, timeVar = "timevisit",
                           method = "piecewise-PH-aGH", parameterization = "slope",
                           derivForm = iform, iter.EM = 250, verbose = TRUE)
summary(jointFit.cum)
```
